# Inherited macular degeneration-associated mutations in CNGB3 increase the ligand sensitivity and spontaneous open probability of cone cyclic nucleotide-gated channels

**DOI:** 10.3389/fphys.2015.00177

**Published:** 2015-06-09

**Authors:** Peter C. Meighan, Changhong Peng, Michael D. Varnum

**Affiliations:** ^1^Department of Integrative Physiology and Neuroscience, Program in Neuroscience, Washington State UniversityPullman, WA, USA; ^2^Center for Integrated Biotechnology, Washington State UniversityPullman, WA, USA

**Keywords:** channelopathy, cyclic nucleotide-gated channels, CNGB3, macular degeneration, gating, single-nucleotide polymorphisms, phototransduction

## Abstract

Cyclic nucleotide gated (CNG) channels are a critical component of the visual transduction cascade in the vertebrate retina. Mutations in the genes encoding these channels have been associated with a spectrum of inherited retinal disorders. To gain insight into their pathophysiological mechanisms, we have investigated the functional consequences of several CNGB3 mutations, previously associated with macular degeneration (Y469D and L595F) or complete achromatopsia (S156F, P309L, and G558C), by expressing these subunits in combination with wild-type CNGA3 in *Xenopus* oocytes and characterizing them using patch-clamp recordings in the inside-out configuration. These mutations did not prevent the formation of functional heteromeric channels, as indicated by sensitivity to block by L-*cis*-diltiazem. With the exception of S156F, each of the mutant channels displayed electrophysiological properties reflecting enhanced channel activity at physiological concentrations of cGMP (i.e., a gain-of-function phenotype). The increased channel activity produced by these mutations resulted from either increased functional expression levels, or increased sensitivity to cyclic nucleotides. Furthermore, L595F increased the spontaneous open probability in the absence of activating ligand, signifying a ligand independent gain-of-function change. In addition to the CNGB3 disease-associate mutations, we characterized the effects of several common CNGB3 and CNGA3 single-nucleotide polymorphisms (SNPs) on heteromeric CNGA3+CNGB3 channel function. Two of the SNPs examined (A3-T153M, and B3-W234C) produced decreased ligand sensitivity for heteromeric CNG channels. These changes may contribute to background disease susceptibility when combined with other genetic or non-genetic factors. Together, these studies help to define the underlying molecular phenotype for mutations relating to CNG channel disease pathogenesis.

## Introduction

Retinal photoreceptors are specialized neuronal cells that convert the absorption of light energy into membrane hyperpolarization. This electrical signal is produced by a phosphodiesterase-mediated reduction in the concentration of cGMP, promoting the closure of outer segment cyclic nucleotide-gated (CNG) channels. The CNG channels participating in this cascade are non-selective cation channels composed of CNGA and CNGB subunits: CNGA1 and CNGB1 (A1+B1) for rod photoreceptors; CNGA3 and CNGB3 (A3+B3) for cone photoreceptors (Craven and Zagotta, 2006; Pifferi et al., 2006). Although CNGA and CNGB subunits share many important structural features—including six putative transmembrane domains, intracellular amino, and carboxyl termini, a cyclic nucleotide binding domain, and a conserved pore region—important functional differences exist between these subunit types. For example, both CNGA1 and CNGA3 can form functional homomeric channels when expressed alone, while CNGB1 and CNGB3 fail to form functional homomeric channels in heterologous expression systems (Chen et al., 1993; Gerstner et al., 2000). In native photoreceptors, CNGB subunits promote the ciliary localization of heteromeric channels (Michalakis et al., 2006; Kizhatil et al., 2009) and profoundly affect several critical biophysical features of the channels including ion selectivity, ligand affinity, ligand discrimination, and spontaneous open probability (Chen et al., 1993; Körschen et al., 1995; Gerstner et al., 2000). Furthermore, the presence of CNGB subunits modulates the sensitivity of CNG channels to putative regulatory factors, such as Ca^2+^-calmodulin, phosphoinsositides, and matrix metalloproteinases (Chen et al., 1994; Brady et al., 2006; Bright et al., 2007; Meighan et al., 2012; Rebrik et al., 2012).

The human *CNGA3* and *CNGB3* genes—which encode for CNGA3 and CNGB3 subunits, respectively—each exhibit a surprising degree of sequence variation. At present, there are reported to be >300 single nucleotide polymorphisms within the coding regions of human *CNGA3* and *CNGB3* (collectively). A majority of these polymorphisms produce an amino acid substitution within the primary structure. Although many identified SNPs have a low frequency of occurrence, others are relatively common; for example, the CNGB3 SNP, T298P, has a reported mean allele frequency (MAP) of ~34%^1^. The specific effects of these genetic variations on CNG channel function and photoreceptor physiology are not well understood. In addition to benign CNG channel sequence polymorphisms, there are currently >110 documented disease-associated mutations in *CNGA3* and *CNGB3* (collectively) (Becirovic and Biel, 2013a,b). These mutations are thought to contribute to the pathogenesis of various hereditary retinal diseases, including macular degeneration, achromatopsia, and progressive cone dystrophy (Kohl et al., 2000, 2004; Sundin et al., 2000; Wissinger et al., 2001; Johnson et al., 2004; Michaelides et al., 2004; Nishiguchi et al., 2005). Most of these mutations are linked to achromatopsia, an autosomal recessive retinal disorder with absent or limited cone photoreceptor function but normal rod function, impaired color vision, reduced visual acuity, congenital photophobia, and nystagmus. Although a greater variety of specific mutations in *CNGA3* have been linked to achromatopsia compared to mutations in *CNGB3*, mutations in *CNGA3* only account for 20–30% of the known cases (Wissinger et al., 2001; Johnson et al., 2004). In contrast, mutations in *CNGB3* are responsible for 40–50% of the cases (Kohl et al., 2004). Although several studies have characterized the functional consequences of cone CNG channels containing disease-associated mutations in CNGA3 and CNGB3 subunits (Peng et al., 2003b; Okada et al., 2004; Tränkner et al., 2004; Bright et al., 2005; Muraki-Oda et al., 2007; Reuter et al., 2008; Duricka et al., 2012; Liu et al., 2013; Tanaka et al., 2014), the range of molecular phenotypes associated with these mutations remains incompletely explored.

In order to better understand the molecular and pathophysiological mechanisms underlying disease-associated mutations in cone CNG channels, we have examined the functional properties of cone CNG channels containing five previously uncharacterized mutations in CNGB3. Two of these mutations [Y469D and L595F (Nishiguchi et al., 2005)], are linked to macular degeneration (MD)/Stargardt's disease, which affects the central regions of the retina leading to a loss of central vision. Three of these mutations [S156F, P309L (Kohl et al., 2004), and G558C (Nishiguchi et al., 2005)] have been linked to achromotopsia. With the exception of S156F, each of the mutant channels examined here produced a gain-of-function phenotype, with the two MD-associated mutations producing the most dramatic changes in CNG channel function.

## Materials and methods

### Molecular biology and functional expression

For heterologous expression in *Xenopus laevis* oocytes, the coding sequences for human CNGA3 (Yu et al., 1996) and CNGB3 (Peng et al., 2003a) were subcloned into pGEMHE (Peng et al., 2004). Site-directed mutagenesis of CNGA3 and CNGB3 was carried out as previously described (Peng et al., 2003a). Oocytes were isolated and microinjected with ~5 ng of mRNA (for all constructs); mRNA concentrations were determined and relative amounts standardized by both denaturing gel electrophoresis and UV spectroscopy. The animal use protocols were consistent with the recommendations of the American Veterinary Medical Association and were approved by the Institutional Animal Care and Use Committee of Washington State University. Each mutant channel construct was examined in at least four independent oocyte injection batches, and compared to wild-type channels within the same injection batch. For efficient generation of heteromeric channels, the ratio of CNGA3 mRNA to CNGB3 mRNA was 1:2.5 (Peng et al., 2004).

### Electrophysiology

One to 7 days after microinjection of mRNA, patch-clamp experiments were performed in the inside-out configuration. Recordings were made at 20–23°C. Voltage control was provided by an Axopatch 200B amplifier (Molecular Devices, Sunnyvale, CA; formerly Axon Instruments); macroscopic current data were acquired using Pulse software (HEKA Elektronik, Lambrecht, Germany) with a sampling frequency of 25 kHz, and low-pass filtered at 10 kHz. Initial pipette resistances were 0.4–0.8 MΩ. From a holding potential of 0 mV, currents were elicited by voltage steps to +80 mV, then to −80 mV, and back to 0 mV. Intracellular and extracellular solutions contained 130 mM NaCl, 0.2 mM EDTA, and 3 mM HEPES (pH 7.2). The cyclic nucleotides, cAMP or cGMP (Sigma-Aldrich, St. Louis, MO), were added to intracellular solutions as indicated. The intracellular solution applied to the face of the patch was changed using an RSC-160 rapid solution changer (Molecular Kinetics, Indianapolis, IN).

### Data analysis

Currents were leak subtracted using the current traces elicited in the absence of cyclic nucleotides before analysis unless otherwise indicated. For channel activation by cGMP dose-response data were fit using the Hill equation: *I*/*I*_MAX_ = [cNMP]*^n^*^H^/(*K*_1/2_*^n^*^H^ + [cNMP]*^n^*^H^), where *I* is the current amplitude, *I*_MAX_ is the maximum current elicited by saturating concentration of ligand, [cNMP] is the ligand concentration, *K*_1/2_ is the apparent ligand affinity, and *n*_H_ is the Hill slope. Fitting with the Hill equation was accomplished with Octave, an open source data-analysis package (www.octave.org), using a custom-fitting routine based on *the method of steepest descent*.

The maximum and spontaneous open probabilities were measured using non-stationary fluctuation analysis (see Alvarez et al., 2002 for review). For the maximum open probability, mean isochrone current variances across 10 current traces were measured for each cGMP concentration. The mean current variance elicited in the absence of cyclic nucleotide was subtracted from the cGMP-elicited variances. Mean variances were plotted against their respective mean macroscopic current amplitudes and were fit with the following parabolic equation:

$$\sigma^{2}=iI-\left(\frac{I^{2}}{N}\right)$$

where σ^2^ is the mean macroscopic current variance, *I* is the mean macroscopic current amplitude, *i* is the single-channel current amplitude, and *N* is the number of conductive channels in the membrane patch. The maximum open probability (*P*_O, MAX_) was calculated using the following relationship:

$$P_{\text{O},\text{MAX}}=\frac{I_{\text{MAX}}}{iN}$$

To calculate the spontaneous open probability (*P*_O, SP_) for wild-type and mutant channels, we used the relationship between the spontaneous macroscopic current variance (i.e., the current variance in the absence of ligand) and the estimated number of channels in the membrane patch (using the relationship above). The macroscopic current variance was determined by the following:

$$\sigma^{2}=Ni^{2}P_{\text{O}}q$$

where *P*_O_ is the open probability, and *q* is the closed probability (and *q* = 1−*P*_O_). Differentiating the current variance with respect to the number of channels *(*dσ^2^*/*d*N)* yields the following quadratic equation:

$$\frac{\text{d}\sigma^{2}}{\text{d}N}=i^{2}P_{\text{O}}\left(1-P_{\text{O}}\right)$$

Solving for *P*_O_ produces an expression for the spontaneous open probability as a function of dσ^2^*/*d*N* and *i*:

$$P_{\text{O},\text{SP}}=\frac{-\sqrt{i^{2}-4\frac{\text{d}\sigma^{2}}{\text{d}N}}-i}{2i}$$

To calculate *P*_O_, we used the average measurements of *i* from fluctuation analyses for each channel type: A3_WT_: *i* = 3.3 pA; A3+B3_WT_: *i* = 3.1 pA; A3+B3_L595F_: *i* = 3.4 pA. The unitary current measurements (at +80 mV) for heteromeric wild-type channels are in reasonable agreement with previous investigations using single-channel recordings under identical conditions (Peng et al., 2003b) or previous investigations of homomeric channels under similar buffering conditions (Yu et al., 1996; Meighan et al., 2012).

### Statistical analysis

ANOVAs, single-pairwise and multiple-pairwise comparisons were performed with *NCSS (www.NCSS.com)*. Data were tested for normality and equal variance prior to hypothesis testing. Single pairwise comparisons of normally-distributed and equal-variant (NDEV) data were analyzed with the Student's *t*-test; multiple pairwise comparisons of NDEV data were analyzed with the Holm-Bonferroni corrected *t*-test (Holm, 1979) following a statistically significant ANOVA result. Data that violated assumptions of normality and equal variance were analyzed with Kruskal-Wallis and Mann-Whitney *U*-tests as indicated. A *p*-value less than 0.05 was considered to be statistically significant for all hypothesis tests. All values are reported as the mean ± S.E.M. of *n* experiments (patches) unless otherwise indicated.

## Results

### Mutant CNGB3 subunits form functional heteromeric CNG channels with CNGA3

**Figure 1:**
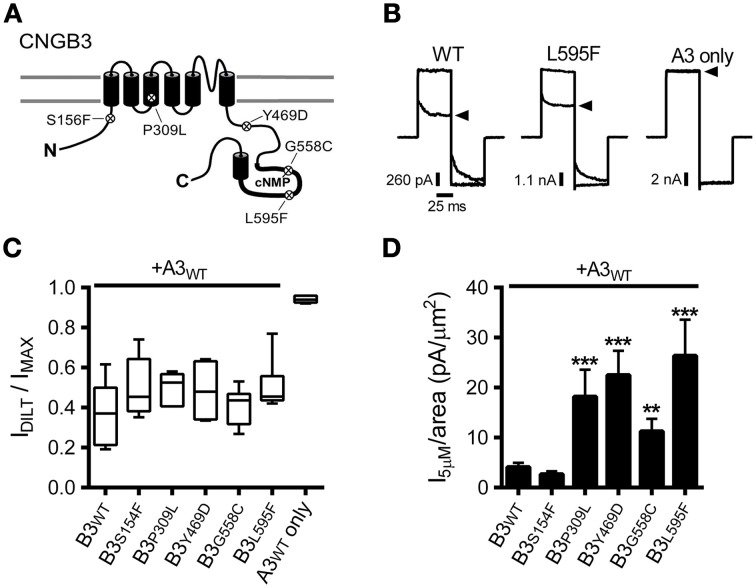
**CNGB3 disease-associated mutations impart a gain-of-function phenotype in heteromeric CNG channels. (A)** Diagram showing CNGB3 subunit topology with approximate locations of disease associated mutations examined in this paper. **(B)** Representative current traces from inside-out patches excised from X*enopus* oocytes expressing heteromeric human A3+B3 channels activated by a saturating concentration (1 mM) of cGMP in the presence (arrowhead) or absence of 25 μM L-cis-diltiazem. Current traces were elicited using voltage steps from a holding potential of 0 mV, to +80 mV, −80 mV, and then returning to 0 mV. **(C)** Box plots summarizing the sensitivity to diltiazem block of channels containing disease-associated mutations compared to wild type A3+B3 heteromeric (WT) and CNGA3-only homomeric channels (A3). Data are expressed as the ratio of the 1 mM cGMP current amplitude at +80 mV in the presence and absence of diltiazem (as in **B**). Boxes represent 25th–75th percentiles, lines show the median, and whiskers represent the 5th–95th percentiles. **(D)** Summary of current densities at 5 μM cGMP for CNGB3 disease-associated mutations. Current-density estimates are based on cGMP dose-response data and estimates of patch area using patch electrode resistance (Sakmann and Neher, 1995). Four of the five mutations examined had an increase in the 5 μM current density compared to wild type A3+B3 channels (*p* < 0.001, single-factor ANOVA, *n* = 6–23; Holm's *t*-test, ^**^p < 0.01, ^***^p < 0.001).

We investigated the functional consequences of five disease-associated mutations in human CNGB3, the cone photoreceptor CNG channel β subunit (Figure 1A). Mutations were introduced into CNGB3 cDNA and these subunits were co-expressed from transcribed cRNA in *Xenopus* oocytes with human cone CNG channel α subunits (CNGA3). Previous reports have characterized CNGB3 mutations that produce non-functional CNGB3 subunits (e.g., T383fsX), which fail to form heteromeric channels when coexpressed with CNGA3, resulting in homomeric CNGA3-only channels (Peng et al., 2003b; Liu et al., 2013). Therefore, we first tested for functional expression of heteromeric channels by application of the CNG channel blocker L-*cis*-diltiazem in the presence of a saturating concentration of cGMP (1 mM). Application of diltiazem to the cytoplasmic face of the membrane blocks native and recombinant heteromeric CNG channels containing CNGB3 (or CNGB1) subunits, but exerts only minimal block on homomeric CNGA3 channels (McLatchie and Matthews, 1992; Chen et al., 1993; Gerstner et al., 2000; Peng et al., 2003a). For each of the mutations examined, application of diltiazem to excised inside-out patches reduced the cGMP-stimulated current amplitude (Figures 1B,C). The level of diltiazem block for the mutant channels was statistically indistinguishable from wild type (WT) heteromeric channels (*p* > 0.05, single-factor ANOVA, *n* = 4–11). This indicates that each of the CNGB3 mutations examined did not interfere with the formation of functional heteromeric A3+B3 channels.

The photoreceptor “dark current” is normally produced by a subsaturating concentration of cGMP (2–5 μM), eliciting a low probability of channel opening (i.e., *P*_O_ < 0.1) (Pugh and Lamb, 1990). Many retinal diseases are thought arise from a deleterious enhancement in CNG channel activity, promoting photoreceptor cell death (Peng et al., 2003b; Bright et al., 2005; Paquet-Durand et al., 2011; Liu et al., 2013). Therefore, we tested whether the disease-associated mutations described above alter the current amplitude relative to the patch area (i.e., the current density) produced by a *subsaturating* concentration of cGMP. For most of the mutant channels examined (P309L, Y469D, G558C, and L595F), we observed a significant increase in the subsaturating current density relative to WT channels (Figure 1D). Therefore, increased CNG channel activity may be a shared feature for channels containing a subset of CNGB3 disease-associated mutations.

The macroscopic current amplitude (*I*) is determined by the single channel current amplitude (*i*), the number of conductive channels (*N*) and the channel open probability (*P*_O_), where *I* = *iNP*_O_. Because each of the mutations examined here are distant from the channel pore-forming region, we expect that the increased subsaturating current densities described above are most likely caused by: (1) increased functional expression of heteromeric A3+B3 channels (increasing *N*); and/or (2) increased sensitivity to cGMP (increasing *P*_O_). To differentiate between these possibilities, first we measured the current density produced by a *saturating* concentration of cGMP (i.e., the maximum current density). The CNGB3 P309L mutation significantly increased the maximum current density compared to wild-type channels (median [25th and 75th percentiles] in pA/μm^2^: WT = 41.0 [18.4, 134.5]; P309L = 206.5 [153.7, 658.0]; *p* < 0.01, Mann Whitney *U, n* = 5–16). The other CNGB3 mutations had no significant effect on the maximum current density (data not shown). The extent by which P309L increased the maximum current density (5.0 times greater than WT) parallels the extent by which the mutation increased the subsaturating current density (4.8 times greater than WT; see Figure 1D). Accordingly, the increased subsaturating current density caused by P309L is likely due to increased functional expression of the mutant channels (i.e., increased *N*).

### Effects of CNGB3 disease-associated mutations on channel ligand sensitivity

**Figure 2:**
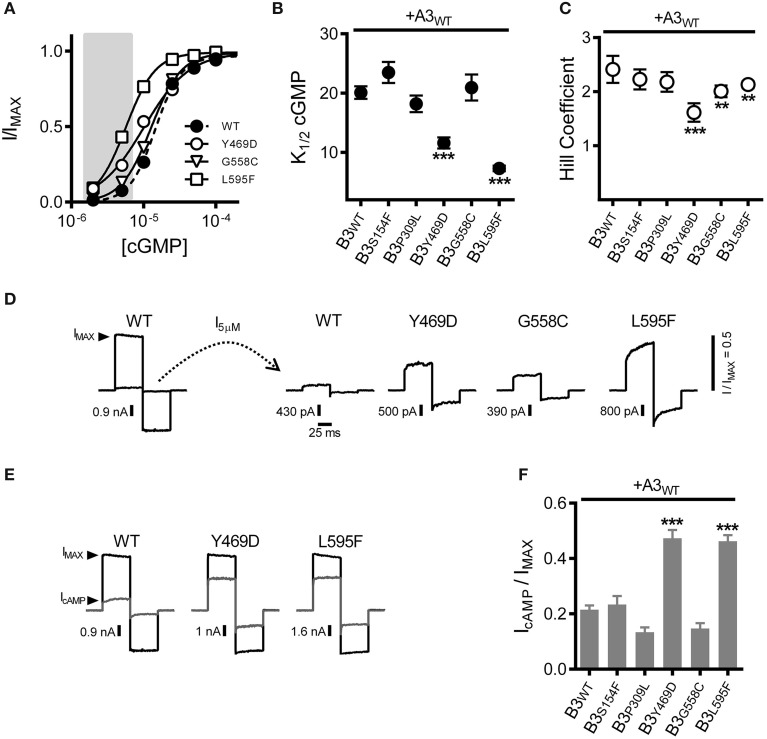
**Macular degeneration-associated Y469D and L595F mutations in CNGB3 each increase the sensitivity of heteromeric A3+B3 channels to physiological ligands. (A)** Representative dose-response curves for activation of A3+B3 wild type (WT; filled circles), A3+B3_Y469D_ (Y469D; open circles), A3+B3_G558C_ (G558C; open triangles), and A3+B3_L595F_ (L595F; open squares) channels by cGMP. Currents were normalized to the maximum cGMP current (*I*_MAX_ = 1.0). Continuous curves represent fits of the dose-response relationship to the Hill equation as indicated in the Materials and Methods. Best-fit parameters are as follows: WT, *K*_1/2_ = 15 μM, *n*_H_ = 2.4; Y469D, *K*_1/2_ = 10 μM, *n*_H_ = 1.4; G558C, *K*_1/2_ = 13 μM, *n*_H_ = 2.1; L595F, *K*_1/2_ = 5.7 μM, *n*_H_ = 2.1. Shaded area represents approximate physiological cGMP concentration in photoreceptors in the dark (Pugh and Lamb, 1993). **(B)** Summary of apparent cGMP affinity (*K*_1/2_) for channels formed by CNGA3 with wild-type CNGB3 or CNGB3 having indicated disease-associated mutations. Data are based on best-fit Hill curves and expressed as mean *K*_1/2_ (± S.E.M.). The *K*_1/2_ cGMP was significantly reduced for the Y469D and L595F groups compared to wild type (*p* < 0.001, Kruskal-Wallis, *n* = 12–22; ^***^p < 0.001, Mann-Whitney *U*-test). **(C)** Summary of mean Hill coefficients (*n*_H_) for channels formed by CNGA3 with wild-type CNGB3 or CNGB3 having disease-associated mutations. The *n*_H_ was significantly reduced for the Y469D, G558C, and L595F groups compared to wild type (*p* < 0.001, single-factor ANOVA, *n* = 5–9; ^***^p < 0.001, ^**^p < 0.01 Holm's *t*-test). **(D)** Representative current traces of heteromeric human A3+B3 channels after activation by a sub-saturating concentration of cGMP (5 μM). Currents were scaled to the maximum current (*I*_MAX_) in a saturating concentration of cGMP (1 mM) as illustrated by the WT current traces. Relative sub-saturating current amplitudes (*I*_5μ*M*_/*I*_MAX_) are as follows: WT = 0.05; Y469D = 0.25; G558C = 0.13; L595F = 0.43. **(E)** Representative current traces after activation by a saturating concentration of cGMP (1 mM, black line) or a saturating concentration of cAMP (10 mM, gray line). **(F)** Summary of relative agonist efficacy (*I*_cAMP_/*I*_cGMP_) for channels containing wild-type CNGB3 or disease-associated mutations, expressed as mean (± S.E.M.). The efficacy of cAMP was significantly enhanced for the Y469D and L595F channels compared to wild type (*p* < 0.001, Kruskal-Wallis, *n* = 5–12; ^***^p < 0.001, Holm's *t*-test).

Results described above suggest that the increased subsaturating current observed for Y469D-, G558C- or L595F-containing channels is not due to altered functional expression levels for heteromeric CNG channels (i.e., not an effect on *N*). Therefore, we tested whether these disease-associated mutations alter the gating properties of A3+B3 heteromeric channels (thereby changing *P*_O_ at subsaturating concentrations of cGMP). First, we examined the effects of the CNGB3 mutations on the apparent cGMP affinity (*K*_1/2_ cGMP) of A3+B3 channels. Dose-response data were collected and fit using the Hill equation. Two of the mutations, Y469D and L595F, each produced a significant decrease in *K*_1/2_ cGMP (i.e., increased apparent affinity) compared to WT channels (Figures 2A,B). Furthermore, Y469D, G558C, and L595F each significantly reduced the Hill coefficient for the cGMP dose-response relationship relative to WT (Figures 2A,C). The reduced Hill coefficients may arise from a change in cooperativity or altered gating properties (e.g., a reduction in the number of activated subunits necessary to promote channel opening) (see Colquhoun, 1998). The increased apparent affinity for cGMP (for Y469D and L595F) and/or reduced Hill coefficient (for Y469D, G558C, and L595F) produced an enhancement in current elicited by 5 μM cGMP, relative to the maximum cGMP stimulated current (*I*_MAX_) for Y469D-, G558C-, and L595F-containing channels (Figure 2D). Additionally, channels containing Y469D and L595F mutations exhibited an increase in the relative agonist efficacy of cAMP—a partial agonist for cone photoreceptor channels (Figures 2E,F). The other mutations examined (S156F and P309L) did not significantly alter channel gating. Collectively, these results suggest that the increased current density for Y469D, G558C, and L595F (see Figure 1D) is likely due to changes in *P*_O_ at subsaturating concentrations of cGMP.

**Figure 3:**
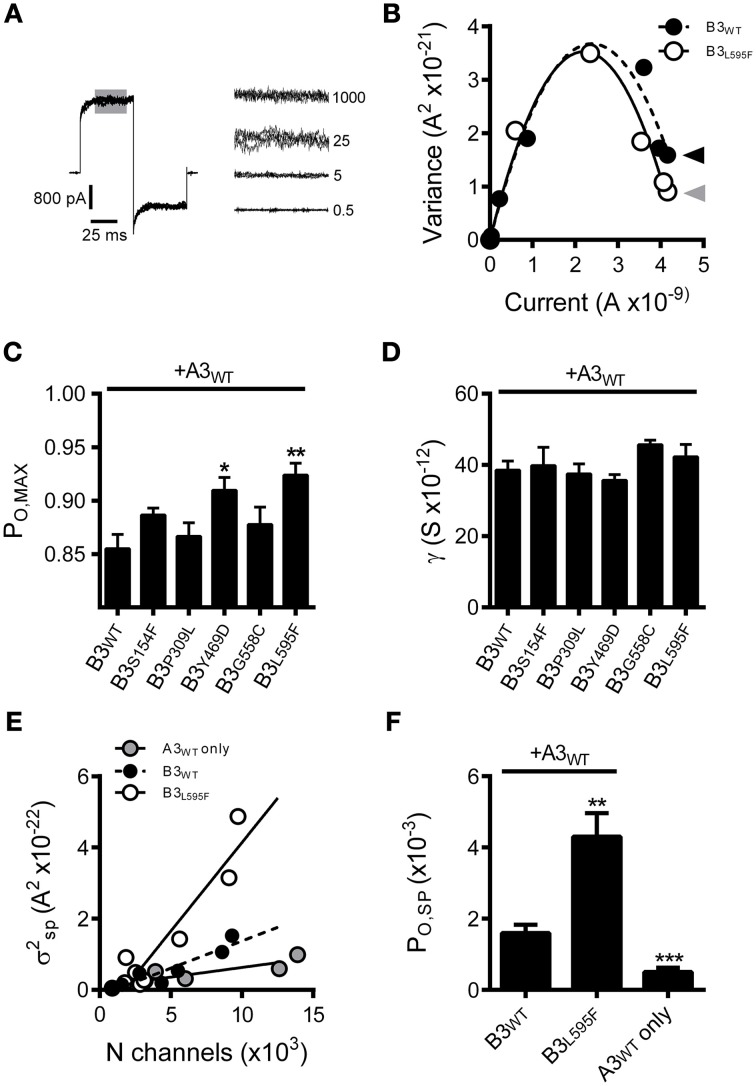
**Effects of CNGB3 disease-associated mutations on the absolute agonist efficacy of cGMP and the spontaneous open probability**. (**A**, *Left*) Overlay of four current traces from A3+B3 channels, activated by a sub-saturating concentration of cGMP (25 μM) to produce an approximately half-maximal current amplitude. Shaded area indicates the region of interest (ROI) used for the calculation of mean current and mean isochrone variance for each concentration of cGMP. (**A**, *Right*) ROI overlays at various cGMP concentrations (μM cGMP indicated on the right). Current and variance calculations were performed as described in the Materials and Methods. The mean isochrone variance is minimal at extreme low and high open probabilities and maximizes at 50% open probability. For wild type cone CNG channels, ~50% open probability is achieved at cGMP concentrations near the *K*_1/2_. The mean isochrone variance at saturating cGMP (i.e., 1 mM) reflects the maximum open probability (*P*_O, MAX_), such that a decreased variance indicates an increased *P*_O, MAX_ (provided that *P*_O, MAX_ > 50%). **(B)** Current-variance plot for A3+B3 wild type (closed circles) and A3+B3_L595F_ (open circles) after activation by cGMP at +80 mV. The L595F mean current and variance estimates were scaled to WT I_MAX_ to facilitate comparison of the initial slope from zero current and the variance at saturating ligand concentrations. Continuous lines produced by fitting with a 2nd order polynomial, as described in the Materials and Methods, using the following best-fit parameters: WT (solid), *i* = 3.1 pA, *N* = 1550 channels; L595F (hashed), *i* = 3.2 pA, *N* = 1370 channels. The mean variance at saturating cGMP is reduced for L595F channels (gray arrowhead) compared to WT channels (black arrowhead), indicating an increased *P*_O, MAX_ for L595F channels. **(C)** Summary of maximal open probabilities after activation with saturating cGMP (1 mM). Maximal open probabilities were calculated as described in the Materials and Methods. The *P*_O, MAX_ was significantly increased for Y469D and L595F channels compared to wild type (*p* < 0.01, single-factor ANOVA, *n* = 4–9; Holm's *t*-test, ^*^p < 0.05, ^**^p < 0.01). **(D)** Summary of unitary conductance values based on best-fit polynomials described in **(B)**. The CNGB3 disease-associated mutations did not significantly alter the unitary conductance (*p* = 0.28; single-factor ANOVA). **(E)** Relationship between the spontaneous isochrone variance (σ^2^_SP_) and estimated number of channels (*N*) in the membrane patch for WT (black circles), L595F (open circles) and A3 only (gray circles) containing channels. The σ^2^_SP_ and *N* estimates were determined as described in the Materials and Methods. Data fit with linear models using the following best-fit slopes (with the units: A^2^/channel x10^−26^): A3+B3 WT = 1.53; A3+B3 L595F = 4.98; A3 WT = 0.58. The slope of the relationship between σ^2^_SP_ and *N* (dσ^2^_SP_/d*N*) was elevated for L595F channels compared to WT, reflecting an increase in the spontaneous open probability (*P*_O, SP_) for L595F channels. **(F)** Spontaneous open probabilities were calculated from trend-line slopes (dσ^2^_SP_/d*N)* from *E*. *P*_O, SP_ calculations were based on the relationship: dσ^2^/d*N* = *i*^2^*Poq*, as described in Materials and Methods. Error estimates of *P*_O, SP_ were propagated from the standard error of slopes from **(E)**. We observed that the spontaneous open probability is significantly lower for A3 homomeric channels (A3_WT_, *P*_O, SP_ = 0.50 ×10^−3^) compared WT heteromeric channels (A3+B3 WT, *P*_O, SP_ = 1.6 x10^−3^) (^***^p < 0.001, extra sum-of-squares *F*-test, *n* = 6, 7). These estimates are in reasonable agreement with previous characterization of mouse cone CNG channels (Gerstner et al., 2000). We also observed that the *P*_O, SP_ is significantly increased for L595F-containing channels (A3+B3 L595F, *P*_O, SP_ = 4.3 ×10^−3^) compared to WT heteromeric channels (^**^p < 0.001, extra sum-of-squares *F*-test, *n* = 7, 8), reflecting a change in the intrinsic gating properties of L595F-containing channels.

To further characterize the effects of these CNGB3 mutations on channel gating, we tested whether the mutations altered the *absolute* agonist efficacies of cGMP and cAMP, as these parameters report channel-gating properties after all ligand-binding sites have been occupied. To address this issue, we determined the maximum open probability (*P*_O, MAX_) in a saturating concentration of cGMP for wild-type and mutant channels using non-stationary fluctuation analysis. Mean currents and isochrone variances were measured and *P*_O, MAX_ was calculated as described in the Materials and Methods (Figures 3A,B). We observed that the *P*_O, MAX_ for CNGB3 mutants Y469D and L595F were increased relative to wild type (Figures 3B,C). Based on these *P*_O, MAX_ values, we estimated the following maximum cAMP stimulated open probabilities (mean *P*_O, cAMP_): WT = 0.18 ± 0.02; Y469D = 0.43 ± 0.03; L595F = 0.42 ± 0.02. Additionally, we used fluctuation analysis to measure the single channel current amplitude of wild type and mutant channels. This method produced estimates of unitary conductance for heteromeric WT channels (γ = 38.5 ± 2.5 pS) that is reasonably consistent with previous studies utilizing single-channel recordings under identical buffer conditions (Peng et al., 2003b). Furthermore, we observed that each CNGB3 mutation did not significantly alter the unitary conductance of heteromeric channels (Figure 3D).

For the Y469D mutation, increased apparent cGMP affinity and cGMP efficacy, concomitant with increased cAMP efficacy, collectively suggests that this mutation may enhance the intrinsic (ligand independent) gating properties of heteromeric CNG channels (for review see Colquhoun, 1998). Interpreting the effects of the L595F mutation on channel gating is problematic, however, because this mutation resides within the cNMP-binding domain; it is plausible that the L595F mutation produces a gain-of-function phenotype by altering ligand-*dependent* and/or the ligand-*independent* gating properties. To address these possibilities, we examined the spontaneous open probability of L595F-containing channels relative to wild-type channels. We expect that if the L595F mutation solely affects ligand dependent gating properties, then the channel's spontaneous open probability (*P*_O, SP_) would be unaffected by this mutation. We analyzed the relationship between the spontaneous current variance (σ^2^_SP_) and the number of functional channels in the membrane patch (*N*). The slope of this relationship (dσ^2^_SP_*/*d*N*) reports the spontaneous open probability, as described in the Materials and Methods. Channels containing CNGB3-L595F subunits exhibited an increase in dσ^2^_SP_*/*d*N* relative to wild-type channels (Figure 3E). Next we used the best-fit slopes of the σ^2^_SP_ and *N* relationships (from Figure 3E) to calculate *P*_O, SP_ for mutant and wild-type channels. This method produced *P*_O, SP_ estimates for wild type human homomeric A3-only (*P*_O, SP_ = 0.50 ×10^−3^) and heteromeric A3+B3 (*P*_O, SP_ = 1.6 × 10^−3^) compositions that are roughly comparable to previous reports of *P*_O, SP_ for channels comprised of mouse CNGA3 and CNGB3 subunits (Gerstner et al., 2000). Furthermore, as indicated by the increase in dσ^2^_SP_*/*d*N* above, we found that the L595F mutation significantly increased spontaneous open probability (*P*_O, SP_ = 4.3 × 10^−3^) (Figure 3F). This finding indicates that the L595F mutation enhances intrinsic (ligand-independent) gating of CNG channels; however, this does not rule out the possibility that additional ligand-specific effects on gating arise from the L595F mutation.

### Effects of CNGB3 single-nucleotide polymorphisms on channel ligand sensitivity

Single nucleotide polymorphisms (SNPs) contribute to the genetic diversity within a species and can affect the susceptibility to disease or disease progression. A number of SNPs have been identified for human *CNGA3* and *CNGB3*, but it is unknown how they affect the functional properties of heteromeric CNG channels. To address this issue, three relatively common CNGB3 SNPs (W234C, T298P, or M466T) were introduced into cDNA encoding the human CNGB3 subunit, and were co-expressed with wild type human CNGA3 subunits in *Xenopus* oocytes. The rationale for selecting these SNPs was based on the following: each of these SNPs has a high frequency of occurrence (i.e., MAP) relative to other SNPs; each site exhibits high sequence conservation across orthologous and parologous sequences; and each SNP represents a non-conservative amino acid change relative to the reference sequence. We also examined one CNGA3 SNP, T153M, in the context of heteromeric channels after co-expression with wild type human CNGB3 subunits.

**Figure 4:**
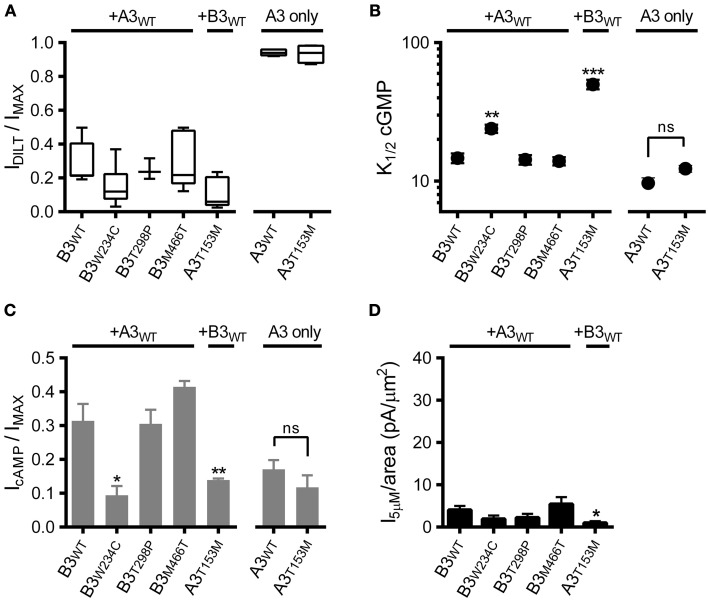
**Effects of single nucleotide polymorphisms on ligand sensitivity of heteromeric A3+B3 channels**. **(A)** CNGA3 and CNGB3 single nucleotide polymorphisms (SNPs) examined here form functional A3+B3 heteromeric channels. Box plots summarizing the sensitivity to block by 25 μM diltiazem in the presence of 1 mM cGMP for SNP-containing channels compared to wild type A3+B3 and A3-only channels. **(B)** Summary of *K*_1/2_ cGMP for wild type and SNP-containing A3 or A3+B3 channels. Data are based on best-fit Hill parameters and expressed as mean *K*_1/2_ (± S.E.M.). The *K*_1/2_ cGMP was significantly increased for the A3+B3_W234C_ and A3_T153M_+B3 groups compared to wild type A3+B3 channels (*p* < 0.001, single-factor ANOVA, *n* = 4–7; Holm's *t*-test, ^**^p < 0.01, ^***^p < 0.001). There was no significant difference between wild-type A3 and A3_T153M_ homomeric channels (Holm's *t*-test, *p* > 0.05). **(C)** Summary of relative agonist efficacy for wild type and SNP-containing A3 or A3+B3 channels. The relative efficacy of cAMP was significantly decreased for A3+B3_W234C_ and A3_T153M_+B3 channels compared to wild type A3+B3 channels (*p* < 0.001, Kruskal-Wallis, *n* = 4–7; Holm's *t*-test, ^*^p < 0.05). There was no significant difference between wild-type A3 and A3_T153M_ homomeric channels (Student's *t*-test, *p* > 0.05). **(D)** Summary of 5 μM cGMP current densities for select CNG channel polymorphisms. The estimated 5 μM current density for heteromeric A3_T153M_+B3 channels was decreased compared to wild type A3+B3 channels (*p* < 0.05, single-factor ANOVA, *n* = 4–8; Holm's *t*-test, ^*^p < 0.05).

We first confirmed expression of heteromeric CNG channels by application of diltiazem (as in Figures 1B,C). Based on the observed sensitivity to diltiazem block (Figure 4A), each of the SNPs examined formed functional heteromeric channels. Next, we assessed the impact of the CNGA3 and CNGB3 SNPs on apparent affinity for cGMP and the relative efficacy of cAMP. Two of the SNPs examined, CNGB3-W234C and CNGA3-T153M, produced a significant increase in *K*_1/2_ cGMP (decreased apparent affinity) for heteromeric channels relative to WT channels (Figure 4B). Consistent with the effects on cGMP apparent affinity, these SNPs also decreased the relative efficacy of cAMP (Figure 4C). Furthermore, the gating effects produced by CNGA3-T153M in heteromeric channels effected a significant reduction in subsaturating current relative to WT (Figure 4D). Other SNPs examined (T298P and M466T) produced no significant changes in the gating properties of heteromeric channels.

**Figure 5:**
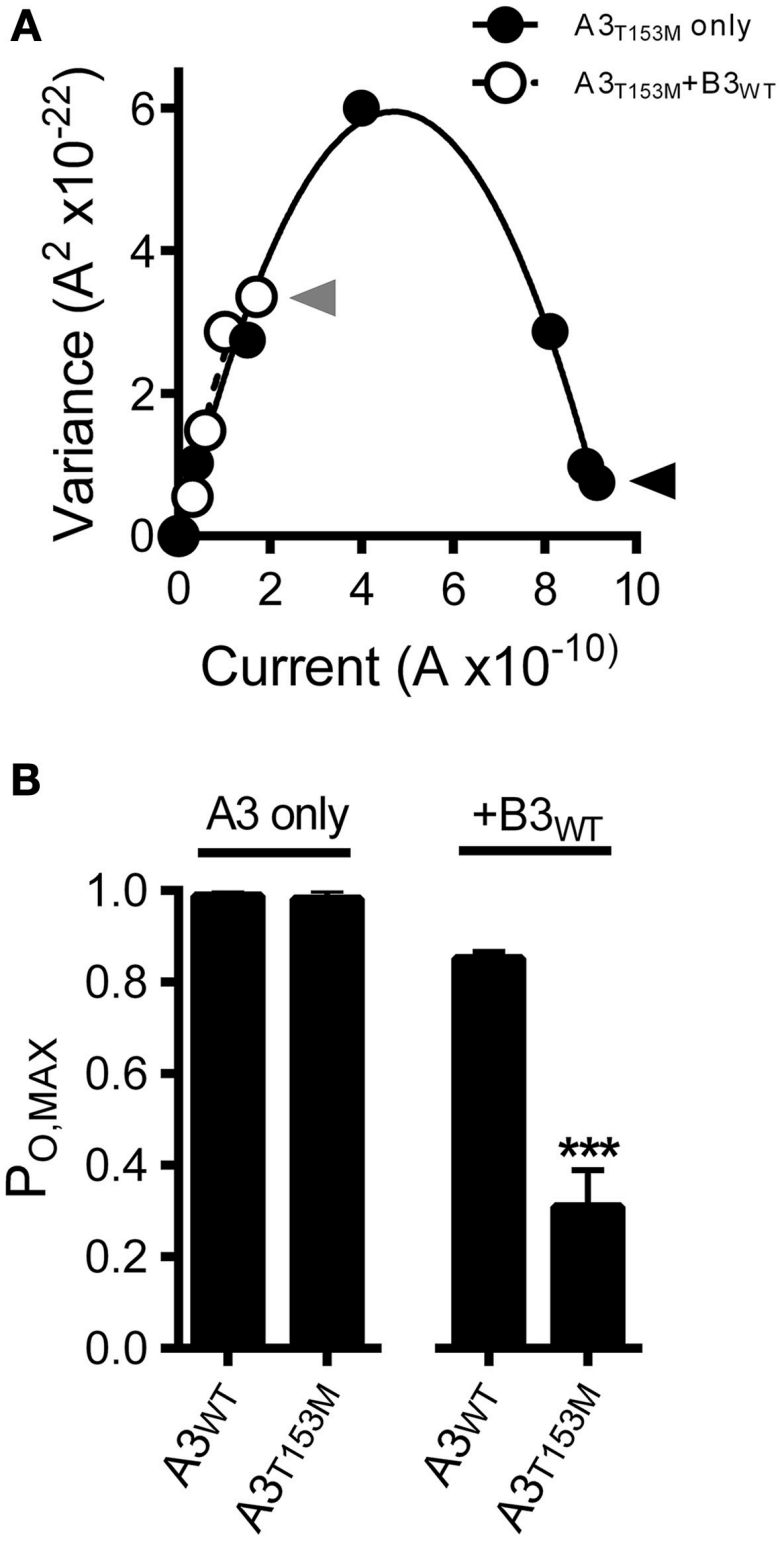
**CNGA3-T153M polymorphism decreases the maximum open probability for heteromeric but not homomeric CNG channels**. **(A)** Current-variance plot for A3_T153M_ homomeric (open circles) and A3_T153M_+B3 (closed circles) channels following activation by various concentrations cGMP at +80 mV (as in Figure 3). Continuous lines were produced by fitting with a 2nd-order polynomial using the following best-fit parameters: A3_T153M_ (solid), *i* = 2.5 pA, *N* = 370 channels; A3_T153M_+B3 (hashed), *i* = 2.5 pA, *N* = 370 channels. Arrows denote mean variance at saturating cGMP for A3_T153M_ (solid) and A3_T153M_+B3 (hashed). **(B)** Maximum open probability was decreased for heteromeric A3_T153M_+B3, but not homomeric A3_T153M_ channels compared to wild-type channels (^***^p < 0.001, Student's *t*-test, *n* = 4–8).

Interestingly, the CNGA3-T153M SNP, which decreased the ligand sensitivity of heteromeric channels, failed to significantly alter the gating properties of homomeric A3-only channels (Figures 4B,C). To further characterize the impact of the CNGA3-T153M SNP on homomeric and heteromeric channel function, we examined the maximum open probability for channels having the CNGA3-T153M SNP relative to reference channel compositions. Mean currents and isochrone variances were measured (Figure 5A) and *P*_O, MAX_ was calculated as described above (see Figure 3). Consistent with the gating effects described above, T153M containing homomeric channels exhibited a *P*_O, MAX_ that was statistically indistinguishable from wild type A3-only channels (Figure 5B); in contrast, T153M containing heteromeric channels exhibited a significant reduction in *P*_O, MAX_ relative to wild-type channels (Figure 5B). Thus, the CNGA3-T153M SNP imparts a profound loss-of-function phenotype that only manifests in the context of A3+B3 heteromeric channels.

## Discussion

**Figure 6:**
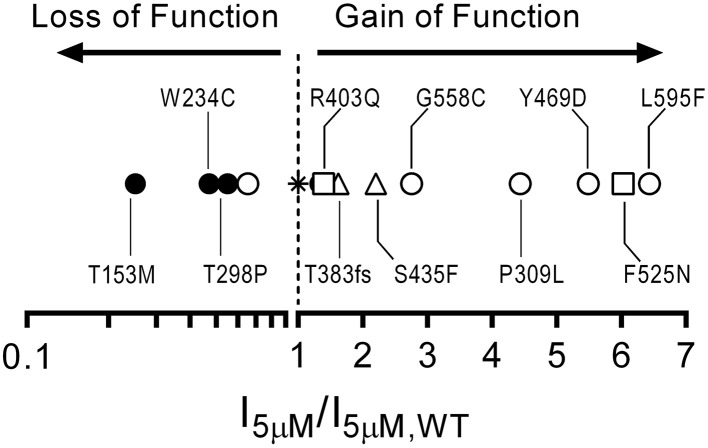
**Gain-of-function/loss-of-function continuum for cone CNG channel disease-associated mutations and polymorphisms based on current densities at a physiological concentration of cGMP**. Plot comparing 5 μM current density ratios for CNGB3 disease associated mutations and CNG channel polymorphisms with wild type A3+B3 channels (star). The plot includes CNGB3 disease-associated mutations described here (open circles) and several CNGB3 disease-associated mutations described previously (Peng et al., 2003b—open triangles; Bright et al., 2005—open squares). Channels with a current-density ratio greater than 1.0 are predicted to generate an elevated photoreceptor dark current compared to wild type A3+B3 channels and thus can be characterized as having a putative gain-of-function phenotype. Conversely, channels with a current-density ratio less than 1.0 are predicted to generate an attenuated photoreceptor dark current compared to wild type channels and can thus be characterized as having a putative loss-of-function phenotype.

We have characterized the functional effects of several disease-associated mutations in CNGB3 (S156F, P309L, Y469D, G558C, and L595F). Most of the mutations examined here produced a gain-of-function phenotype, causing increased current levels at physiological concentrations of cGMP (Figure 6). This finding is consistent with the hypothesis that abnormally elevated CNG channel activity is causally linked to photoreceptor dysfunction and retinal degeneration (Paquet-Durand et al., 2011; Liu et al., 2013). Additionally, we described the functional effects of common single nucleotide polymorphisms for CNGB3 (W234C, T298P, and M466T) and CNGA3 (T153M). In contrast to the disease-associated mutations, the SNPs examined here either failed to impact channel function, or produced a loss-of-function phenotype, resulting in decreased current levels at physiological concentrations of cGMP (Figure 6). These results demonstrate that common SNPs in CNG channel genes can alter channel function, and thus may influence disease susceptibility and progression in the context of other mutations or environmental factors.

There are several mechanisms that may promote a deleterious elevation in CNG channel activity in photoreceptors. For the CNGB3 mutations examined here, we observed increased subsaturating current by three mechanisms. First, the achromatopsia-associated mutation, P309L, produced a gain-of-function phenotype by increasing functional expression levels in oocytes. It is unknown whether the increased channel density caused by P309L is due to increased trafficking to the plasma membrane, or decreased turnover of channel protein. Although this result is consistent with the presumed connection between CNG channel hyperactivity and photoreceptor cell death, extrapolation from heterologous expression systems to photoreceptors requires discretion. Furthermore, we cannot rule out the possibility that P309L contributes to the etiology of achromatopsia by altering other photoreceptor-specific processes, such as adaptive changes in channel sensitivity. Further studies are needed to determine how this mutation promotes increased channel surface expression, and whether this result translates to altered expression levels in photoreceptors. Second, two macular degeneration/Stargardt's disease-associated mutations, Y469D and L595F, each produced a gain-of-function phenotype by altering CNG channel gating. Channels containing either Y469D or L595F demonstrated increased sensitivity to their endogenous ligands; thus, increased open probability is expected at physiological concentrations of cGMP. Third, the achromatopsia associated G558C mutation produced a gain-of-function phenotype by reducing the Hill coefficient of the dose-response relationship for cGMP. Although this result could not be attributed to changes in the apparent affinity for cGMP, a reduced Hill coefficient increases channel activity at cGMP concentrations below the *K*_1/2_. Similarly, the reduced Hill coefficient for the Y469D and L595F mutations contributes to the gain-of-function phenotype observed with these mutations. Since photoreceptor cGMP concentrations are thought to be about 2–5 μM in the dark (Pugh and Lamb, 1993), changes in CNG channel responses below the *K*_1/2_ cGMP are expected to be physiologically important.

In addition to these ligand dependent changes in channel function, we observed an increase in the spontaneous (i.e., unliganded) open probability for L595F-containing channels compared to wild-type channels. This result is intriguing considering that CNG channel *closure* is a critical event within the phototransduction cascade. This suggests that CNGB3 mutations could plausibly interfere with channel deactivation during light stimulation and contribute to the background conductance in the photoreceptor—a ligand-independent, gain-of-function change. Furthermore, this ligand-independent effect was somewhat unexpected, considering that L595F resides within the CNBD. How might a mutation within the CNBD exert a global effect on channel gating? L595 represents a highly conserved leucine located within the phosphate binding cassette (PBC) of the canonical cyclic nucleotide binding domain; the PBC interacts with phosphate and ribose moieties of the cyclic nucleotide upon ligand binding. The increase in the spontaneous open probability suggests that L595 influences the allosteric transition underlying channel opening and that the L595F mutation stabilizes the open state relative to the closed state. Based on NMR spectroscopy of *Mesorhizobium* loti K1 (MloK1) channels and double electron–electron resonance (DEER) spectroscopy of HCN channels, the PBC participates in the allosteric conformational change within the CNBD (Schünke et al., 2011; Puljung et al., 2014). Interestingly, NMR spectroscopy indicates that the homologous leucine residue within the PBC of MloK1 (L301) is significantly displaced upon ligand binding. The authors speculate that in the absence of ligand, the L301 side chain contributes to a hydrophobic pocket that sterically prevents the B and C helices of the CNBD from assuming the ligand-bound position. Ligand binding promotes the repositioning of L301 thus permitting the B and C helices to shift toward the β roll during the allosteric conformational change. NMR spectroscopy and DEER measurements of conformational rearrangements in the CNBD of HCN2 also imply repositioning of the PBC (Puljung et al., 2014; Saponaro et al., 2014). Assuming L595 in CNGB3 similarly contributes to the gating movements in the CNBD, we speculate that the leucine-to-phenylalanine mutation exerts ligand-independent effects on the conformation of the PBC and/or the B and C helices, which make this allosteric transition more favorable.

In the context of photoreceptor function, the mutations producing a gain-of-function phenotype described above (see Figure 6) are expected to enhance CNG channel activity in the dark (i.e., increase the “dark current”). CNG channels provide the main calcium entry pathway for photoreceptor outer segments (Fain et al., 2001; Korenbrot and Rebrik, 2002). Additionally, it has been demonstrated that hyperactivity of CNG channels—either due to elevated basal cGMP levels (from loss of phosphodiesterase activity or increased guanylate-cyclase activity) or increased channel sensitivity to cGMP—promotes photoreceptor cell death via calcium-dependent mechanisms (Huang et al., 1995; Payne et al., 1998; Biel and Michalakis, 2007; Paquet-Durand et al., 2011; Liu et al., 2013). Consistent with this idea, CNG channel knockout or knockdown can rescue photoreceptors in the context of mutations producing elevated cGMP levels (Paquet-Durand et al., 2011; Tosi et al., 2011). In addition, CNG channel block using L-*cis*-diltiazem can in several scenarios rescue cell viability in the context of disease-associated perturbations (Frasson et al., 1999; Vallazza-Deschamps et al., 2005; Liu et al., 2013). Thus, CNG channels may represent a logical target for intervention in some forms of retinal degeneration.

In contrast to the functional perturbations described for the other disease-associated mutations, S156F produced no significant change in CNG channel behavior. The S156F mutation has been reported in multiple families having the clinical features of achromatopsia (Kohl et al., 2004). S156F may affect some feature other than the basal channel activity measured here, such as mRNA splicing or protein stability in photoreceptors. S156F also might influence channel regulation by second messengers, as it represents a potential phosphorylation site and is adjacent to the N-terminal calmodulin-binding site in human CNGB3 (Peng et al., 2003a). Furthermore, S156 is located within a region of CNGB3 that is not highly conserved (the cytoplasmic N-terminal domain); this residue is present in many but not all orthologous sequences. It remains possible that S156F represents a non-pathogenic sequence variation, perhaps linked to an unknown sequence change in a non-protein coding region of *CNGB3* that might alter mRNA expression levels, processing or stability.

We observed that the SNP T153M in CNGA3 decreased the ligand sensitivity of heteromeric A3+B3 channels but not homomeric A3-only channels. This finding is relevant as this mutation was observed in the genetic analyses of two patients (sisters) diagnosed as incomplete achromats (Kohl et al., 1998; Tränkner et al., 2004). The gating effects produced by T153M were surprising as it had been previously noted that this sequence change does not alter the gating properties of CNG channels (Tränkner et al., 2004). This conclusion was likely based on the previous characterization of T153M being restricted to homomeric (A3 only) channels; similarly, we observe no effect of T153M on homomeric channel function. How might T153M alter the gating properties of A3+B3 heteromeric, but not A3 homomeric channels? One possibility is that T153M alters A3/B3 intersubunit interactions in heteromeric channels, but not A3/A3 interactions in homomeric channels. Intersubunit interactions modulate the ligand sensitivity of CNG channels, and are regulated by a variety of signaling effectors, including Ca^2+^-calmodulin (Trudeau and Zagotta, 2002; Zheng et al., 2003) and phosphoinositides (PIP*_n_*) (Brady et al., 2006; Dai and Varnum, 2013; Dai et al., 2013). Furthermore, the interactions between subunits—and the modulation of intersubunit interactions by signaling effectors—can depend on channel composition (i.e., homomeric vs. heteromeric). For example, it has been demonstrated recently that PIP*_n_*-mediated regulation of cone CNG channel ligand sensitivity requires regulation modules located within CNGA3 subunits, but the competence of those regulation modules depends on assembly of CNGA3 with CNGB3 subunits in heteromeric channels (Dai et al., 2013). This is likely due to differences in intersubunit coupling between N- and C-terminal cytoplasmic domains of A3/B3 subunits vs. A3/A3 subunits (Dai and Varnum, 2013; Dai et al., 2013). Whether the gating effects produced by T153M are due to altered intersubunit coupling in heteromeric channels, and whether this substitution contributes to the retinal pathogenesis, are questions that necessitate future investigations.

## Author contributions

PM, CP, and MV each designed the experiments, performed the research, analyzed data, and wrote the manuscript.

### Conflict of interest statement

The authors declare that the research was conducted in the absence of any commercial or financial relationships that could be construed as a potential conflict of interest.

## Footnotes

^1^NCBI Reference Assembly: CNGB3 SNP Available online at: http://www.ncbi.nlm.nih.gov/projects/SNP/snp_ref.cgi?geneId=54714 [Accessed August 11, 2014].
